# Sweet apple e-cigarette vapor differentially modulates the mTOR pathway in oral squamous cell carcinoma cell lines

**DOI:** 10.3389/fonc.2026.1886799

**Published:** 2026-07-21

**Authors:** Kristina Vu, Logan Ponder, Ryan Kinney, Ankita Chatterje, Harishma Sidhu, Neha Patel, Benjamin T. Bikman, Paul R. Reynolds, Juan A. Arroyo

**Affiliations:** 1 College of Dental Medicine, Roseman University of Health Sciences, South Jordan, UT, United States; 2 Department of Cell Biology and Physiology, Brigham Young University, Provo, UT, United States; 3 Brigham Young University School of Medicine, Provo, UT, United States

**Keywords:** oral squamous cell carcinoma, mTOR, AKT, electronic cigarette, cell invasion

## Abstract

**Objectives:**

Oral squamous cell carcinoma (OSCC) is the most common head and neck cancer and is associated with high recurrence and poor prognosis. This study investigated the effects of Sweet Apple e-cigarette vapor extract (Apple EVE), with and without nicotine, on mTOR pathway activation in OSCC cell lines Ca9–22 and Cal 27.

**Methods:**

Cells were exposed for 6 hours to 10% Apple EVE generated from “Reds Apple Juice” in the presence or absence of nicotine (6 mg), with untreated cells as controls. Phosphorylation levels of mTOR pathway components (p-mTOR, p-p70S6K, p-4EBP1, and p-AKT) were assessed by Western blot and quantified by densitometry normalized to β-actin. Cell invasion was evaluated using a Matrigel-coated real-time xCELLigence assay. Data were analyzed using the Mann–Whitney U test (p < 0.05).

**Results:**

Nicotine-containing Apple EVE significantly increased p-mTOR in both cell lines. In Ca9–22 cells, it decreased p-p70S6K and p-4EBP1, whereas in Cal 27 cells it increased p-AKT. Apple EVE without nicotine induced more modest and variable effects. No significant changes in cell invasion were observed in either cell line.

**Conclusions:**

Apple EVE, particularly when combined with nicotine, differentially modulates mTOR signaling in a cell line-specific manner in OSCC cells. These findings highlight the complex effects of flavored e-cigarette aerosols on cancer-related pathways and warrant further investigation into the functional consequences of these early signaling changes.

## 1 Introduction

Oral squamous cell carcinoma (OSCC) represents the predominant form of head and neck cancer, contributing to approximately 90% of cases within the oral cavity and affecting around 600,000 individuals annually worldwide (1). Despite advancements in therapeutic approaches, OSCC is marked by a high propensity for early metastasis and a recurrence rate of up to 50%, resulting in poor prognosis for many patients (2, 3). The primary risk factors for OSCC include tobacco use and excessive alcohol consumption, but emerging evidence suggests that electronic cigarette (e-cigarette) use may also play a significant role in oral carcinogenesis (4). Unlike traditional cigarette smoke, e-cigarette aerosols deliver a complex mix of chemicals, including nicotine and flavoring agents, directly to the oral mucosa, potentially amplifying carcinogenic effects (5). The rising prevalence of e-cigarette use, particularly among adolescents and young adults, with usage rates ranging from 16–28% in these groups, underscores the urgency to investigate their impact on oral health (6, 7). Fruit-flavored e-cigarettes, including apple-based formulations, represent some of the most commonly used products among adolescents and young adults and are frequently perceived as less harmful than traditional tobacco products. The Sweet Apple formulation used in this study was selected due to its popularity among users and prior evidence from our laboratory demonstrating its capacity to modulate inflammatory and invasion-related pathways in oral squamous cell carcinoma cells (1), thereby providing a relevant and mechanistically informative exposure model.

The mammalian target of rapamycin (mTOR) pathway is a critical regulator of cellular processes, including proliferation, metabolism, and survival, and its dysregulation is implicated in various malignancies, including OSCC (8, 9). Key downstream effectors of mTOR, such as p70S6 kinase (p70S6K) and eukaryotic translation initiation factor 4E-binding protein 1 (4EBP1), mediate protein synthesis and cell growth (10). Prior studies have shown that inhibition of mTOR signaling can induce apoptosis and reduce tumor cell migration, suggesting its therapeutic potential (11). Our previous research demonstrated that e-cigarette vapor, particularly flavored formulations such as sweet apple with varying nicotine concentrations, enhances OSCC cell invasion and inflammatory responses (1).

Oral squamous cell carcinoma is a heterogeneous disease characterized by substantial variability in genetic background, signaling pathway utilization, and therapeutic response (1, 11). Consequently, environmental exposures may elicit distinct molecular responses across different OSCC cell lines. The present study was designed to evaluate early, cell-line–dependent perturbations in the mTOR signaling axis following exposure to flavored e-cigarette vapor extract, rather than to define a single uniform mechanistic pathway. By examining key nodes within the mTOR pathway in two biologically distinct OSCC cell lines, this work aims to provide hypothesis-generating insights into how flavored e-cigarette aerosols may differentially modulate cancer-associated signaling pathways in oral epithelial malignancies.

## 2 Materials and methods

### 2.1 Cell culture

Ca9–22 human oral squamous carcinoma cells and Cal 27 human tongue squamous carcinoma cells (both from ATCC, Manassas, VA) were used in these experiments. Cells were maintained in RPMI medium plus 10% fetal bovine serum (FBS) (Invitrogen, Carlsbad, CA, USA).

### 2.2 Sweet apple eCig vapor extract

EVE was generated as previously shown by our laboratory (1). Briefly, an eCig module was connected, by the mouthpiece, to a vacuum pump while pressing the button on the eCig module for 3 seconds. The vacuum pump drew vapor through the tip of a pipette submerged in a tube containing 10 mL of serum-free medium. This process was repeated using 3-second puffs, each followed by a 20-second rest, for a total of 20 puffs, and the conditioned medium was identified as the 100% EVE solution. This procedure was performed using Reds Apple Juice (Green Apple, Daze Mfg., Los Angeles) in the presence or absence of 6 mg of nicotine and compared to untreated cells (control). Although precise nicotine quantification was not analytically measured, dilution parameters were kept constant across experiments to ensure reproducibility. The 6 mg designation refers to the manufacturer-labeled nicotine content of the e-liquid rather than an analytically confirmed delivered dose; mass spectrometric quantification of nicotine in the vapor extract was not performed and is acknowledged as a limitation. Vapor generation parameters, including puff duration (3 seconds), number of puffs (20 per preparation), and media volume, were standardized for all experiments to minimize batch-to-batch variability. Vapor was generated using a refillable e-cigarette module with vacuum-assisted extraction rather than a wattage-controlled vaping machine; accordingly, device output power and coil resistance were not independently set, and the exposure is defined by the puff protocol and media volume described above.

### 2.3 Cell treatments

At 80% cell confluency, the two cell lines were incubated for 6 hours in medium alone (control) or medium supplemented with Apple EVE (10%) in the presence or absence of 6 mg nicotine. After the exposure, total cell lysates were obtained.

### 2.4 Western blot

We carried out Western blotting (n=10), adhering to methods previously described by our group. Here, n = 10 denotes independent biological replicates (separate cultures, treatments, and lysates) rather than technical replicates of a pooled lysate (1). To summarize, the cells underwent lysis with RIPA buffer (RIPA, Fisher Scientific, Saint Louis, MO). Extracts containing 30–35 µg of protein were electrophoresed on precast Mini-PROTEAN TGX gels (Bio-Rad Laboratories, Hercules, CA) before being blotted onto nitrocellulose. Overnight probing involved primary antibodies specific for phosphorylated forms of mTOR (Ser2448), p70 (Thr389), 4EBP1 (Thr47/46), and AKT (Ser473; Cell Signaling Technology, Danvers, MA). This was succeeded the following day by a 60-minute exposure to secondary antibodies conjugated with fluorophores. Blot visualization took place via the Li-COR Odyssey CLx imager. We employed a β-actin antibody to normalize for loading variations. Intensity of fluorescent signals was assessed and computed through ImageJ, enabling evaluation of differences among the experimental and baseline samples. Equal protein loading was confirmed by total protein quantification prior to electrophoresis and by normalization to β-actin. Densitometric analyses represent averaged values from independent biological experiments performed under identical conditions, thereby minimizing technical variability. For each phosphoprotein target, membranes were stripped and reprobed on the same blot, and each densitometric graph represents n = 10 independent blots rather than multiple lanes from a single representative blot. 

### 2.5 Real-time cell invasion

We assessed the real-time invasive behavior of OSCC cell lines (*n* = 10) using a previously validated method established by our laboratory (1). After completing the experimental treatments, invasion dynamics were monitored with an xCELLigence RTCA DP instrument (ACEA Biosciences, Blue Springs, MO, USA) equipped with 16-well CIM-Plates. Each well was precoated with a 1:40 dilution of Matrigel (Fisher Scientific, Pittsburgh, PA) to mimic the extracellular matrix barrier. A total of 20,000 cells suspended in 100 µL of RPMI containing 2% FBS were seeded into the upper chamber, while the lower chamber received 160 µL of complete medium supplemented with 10% FBS to provide a chemoattractant gradient. Electrical impedance was recorded automatically at 15-minute intervals for 24 hours. The Cell Index (CI), a dimensionless parameter reflecting real-time changes in cell adhesion, morphology, number, and invasion across the Matrigel-coated electrodes, was automatically calculated by the RTCA software as (impedance at time t – background impedance)/background impedance factor. Invasion indices were derived directly from the RTCA software to quantify cell movement across Matrigel barrier. For the invasion assay, n = 10 likewise refers to independent biological replicates (separate cultures and treatments) rather than technical repeats.

### 2.6 Statistical analysis

**Figure 1:**
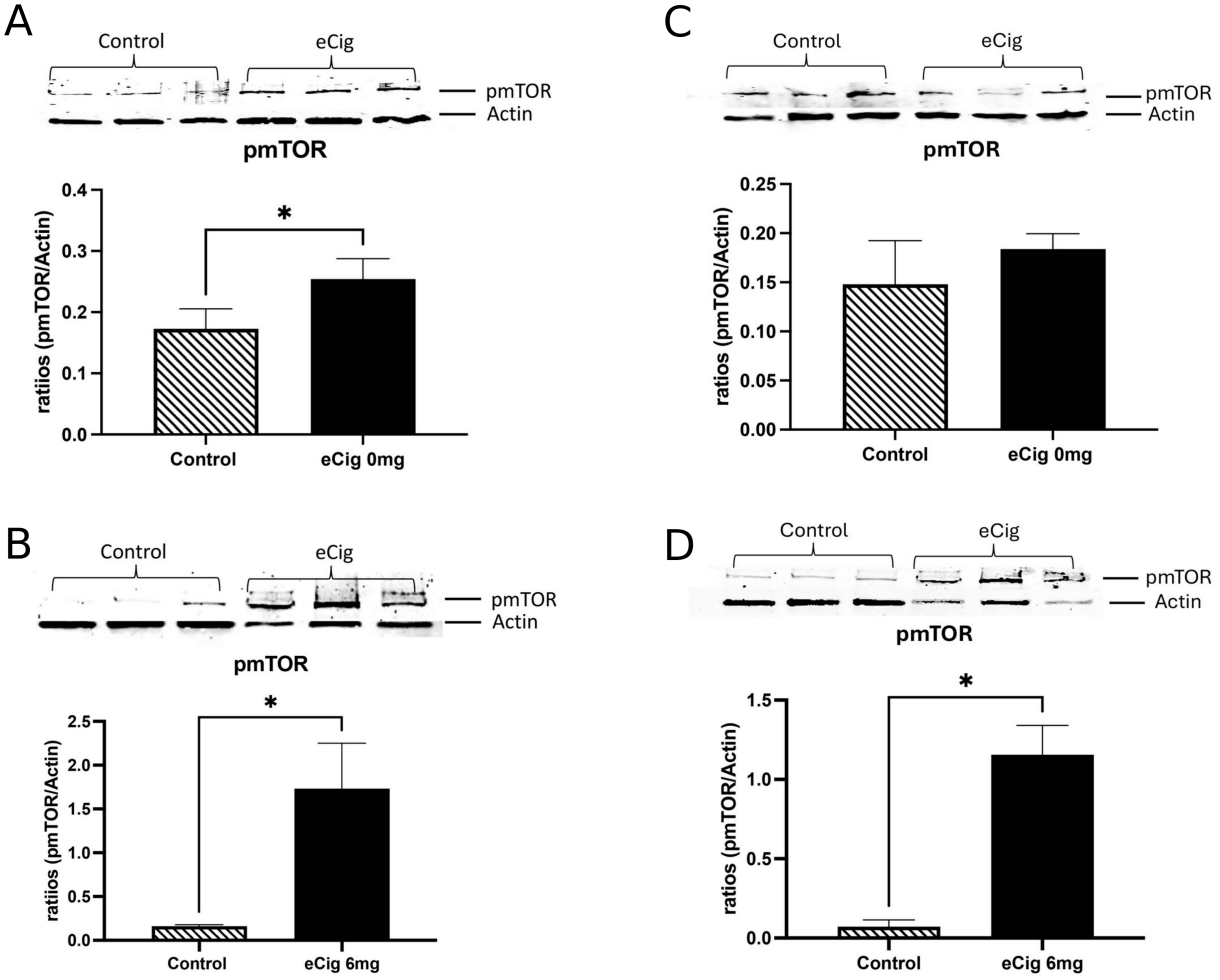
p-mTOR (Ser2448) in OSCC cells treated with Apple EVE. Western blot analysis and densitometric quantification of p-mTOR normalized to β-actin in Ca9–22 and Cal 27 cells following 6 h exposure to 10% Apple EVE with or without nicotine (6 mg). **(A)** Ca9-22, 0 mg nicotine; **(B)** Ca9-22, 6 mg nicotine; **(C)** Cal 27, 0 mg nicotine; **(D)** Cal 27, 6 mg nicotine. Data are presented as mean ± SEM ratios of p-mTOR/β-actin. *p ≤ 0.05 vs. control.

Data are presented as mean ± SEM and represent independent biological replicates. Each condition was tested in n = 10 independent biological experiments, and the Western blots shown are representative of these replicates. Normality of distribution was assessed prior to analysis. The non-parametric Mann–Whitney U test was chosen because it does not assume normality or equality of variance between groups, which is appropriate given the small sample sizes and the unequal variances observed between control and treated conditions (e.g., **Figure 1**). Because comparisons were conducted between predefined experimental groups and controls for each protein independently, data were analyzed as unpaired comparisons using the Mann–Whitney U test, with each phosphoprotein compared against its own matched, concurrent control. A p-value < 0.05 was considered statistically significant. Given the exploratory mechanistic nature of the study and independent evaluation of distinct signaling endpoints, formal correction for multiple testing across different proteins was not applied. A total of 16 comparisons were performed (four phosphoproteins in two cell lines, each with and without nicotine); accordingly, the individual p-values reported should be interpreted as exploratory and hypothesis-generating rather than confirmatory. All statistical analyses were conducted using GraphPad Prism version 8.0 (GraphPad Software, San Diego, CA, USA).

## 3 Results

**Figure 2:**
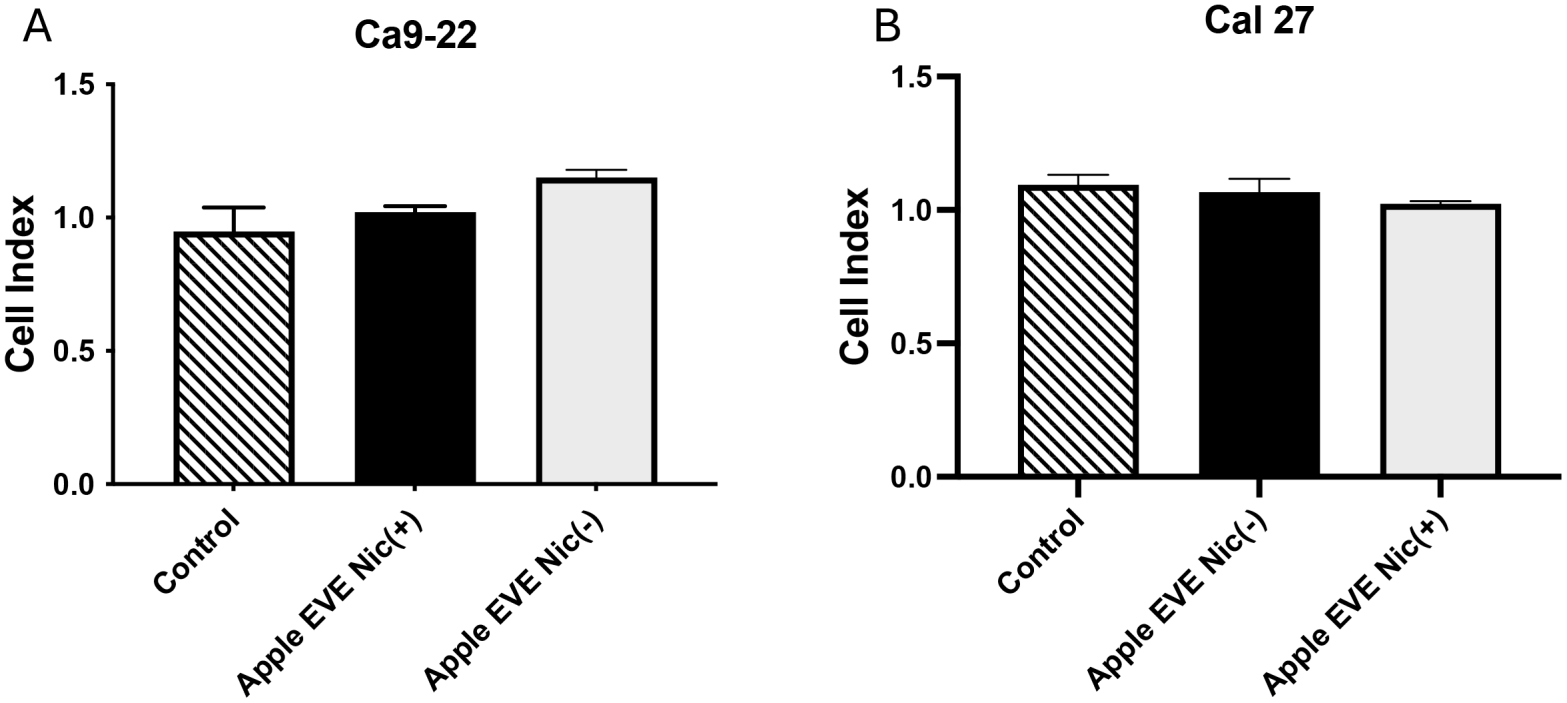
Ca9-22 and Cal 27 invasion with Apple EVE. Apple EVE did not affect Ca9-22 **(A)** nor Cal 27 **(B)** invasion with or without nicotine when compared to controls.

We previously reported no differences in invasion in cells treated with EVE in the absence of nicotine (1). This was confirmed for these experiments, showing no differences in cell invasion for Ca9–22 and Cal 27 when treated with Apple EVE (**Figure 2**). The mTOR protein is a key regulator of cell survival and progression (11). Apple EVE without nicotine increased p-mTOR (1.5-fold; p<0.02) in Ca9–22 cells, while no significant change was observed in Cal 27 cells (**Figures 1A, C**). Apple EVE with nicotine (6 mg) significantly increased p-mTOR in both Ca9-22 (10.8-fold; p<0.01) and Cal 27 (16.1-fold; p<0.001) cells (**Figures 1B, D**). The large fold-changes in the nicotine condition arise in part from low control baseline values, and we therefore emphasize the direction and reproducibility of these effects rather than their absolute magnitude.

**Figure 3:**
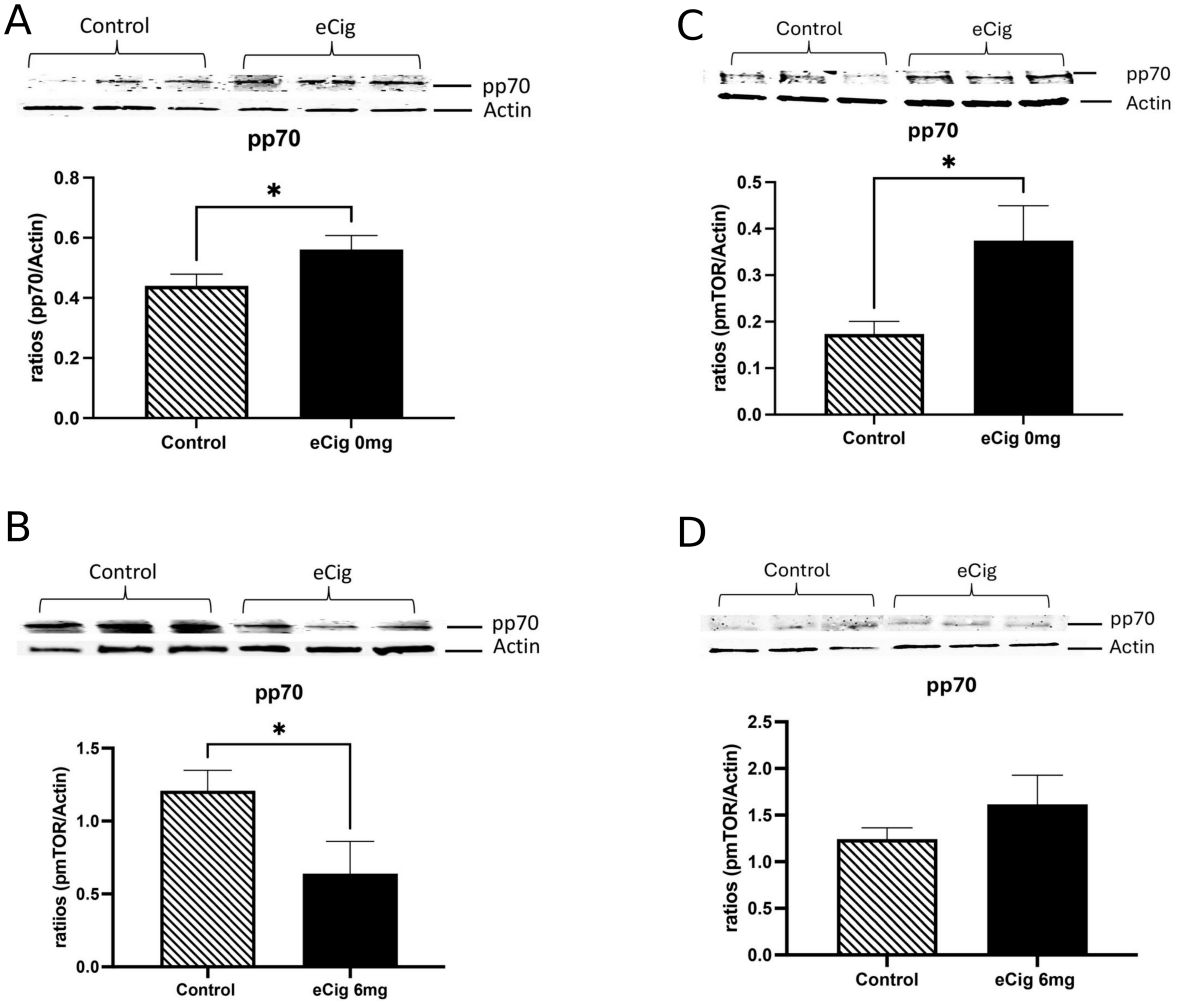
p-p70S6K (Thr389) in OSCC cells treated with Apple EVE. Western blot analysis and densitometric quantification of p-p70S6K normalized to β-actin in Ca9–22 and Cal 27 cells following 6 h exposure to 10% Apple EVE with or without nicotine (6 mg). **(A)** Ca9-22, 0 mg nicotine; **(B)** Ca9-22, 6 mg nicotine; **(C)** Cal 27, 0 mg nicotine; **(D)** Cal 27, 6 mg nicotine. Data are presented as mean ± SEM ratios of p-p70S6K/β-actin. *p ≤ 0.05 vs. control.

**Figure 4:**
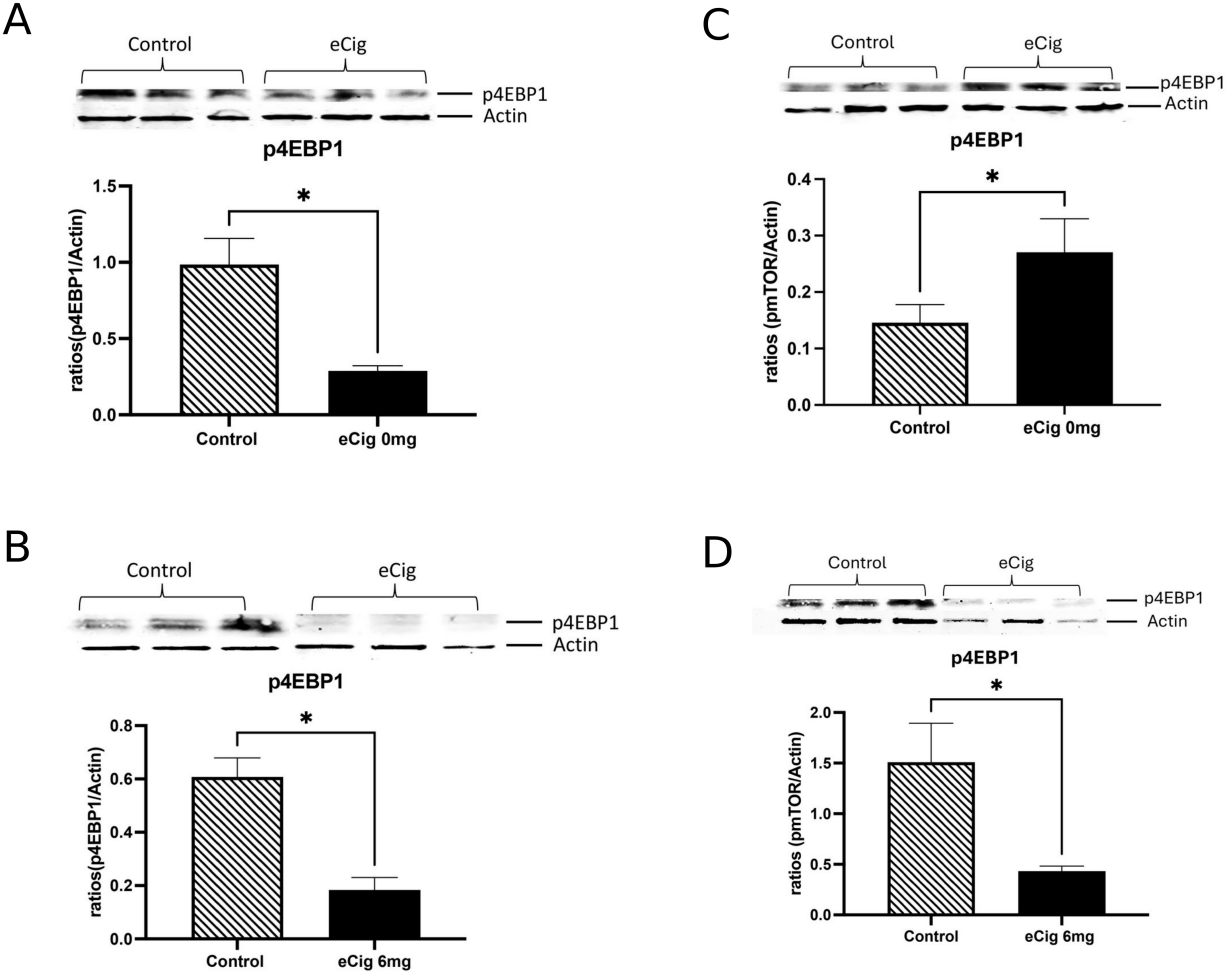
p-4EBP1 (Thr37/46) in OSCC cells treated with Apple EVE. Western blot analysis and densitometric quantification of p-4EBP1 normalized to β-actin in Ca9–22 and Cal 27 cells following 6 h exposure to 10% Apple EVE with or without nicotine (6 mg). **(A)** Ca9-22, 0 mg nicotine; **(B)** Ca9-22, 6 mg nicotine; **(C)** Cal 27, 0 mg nicotine; **(D)** Cal 27, 6 mg nicotine. Data are presented as mean ± SEM ratios of p-4EBP1/β-actin. *p ≤ 0.05 vs. control.

**Figure 5:**
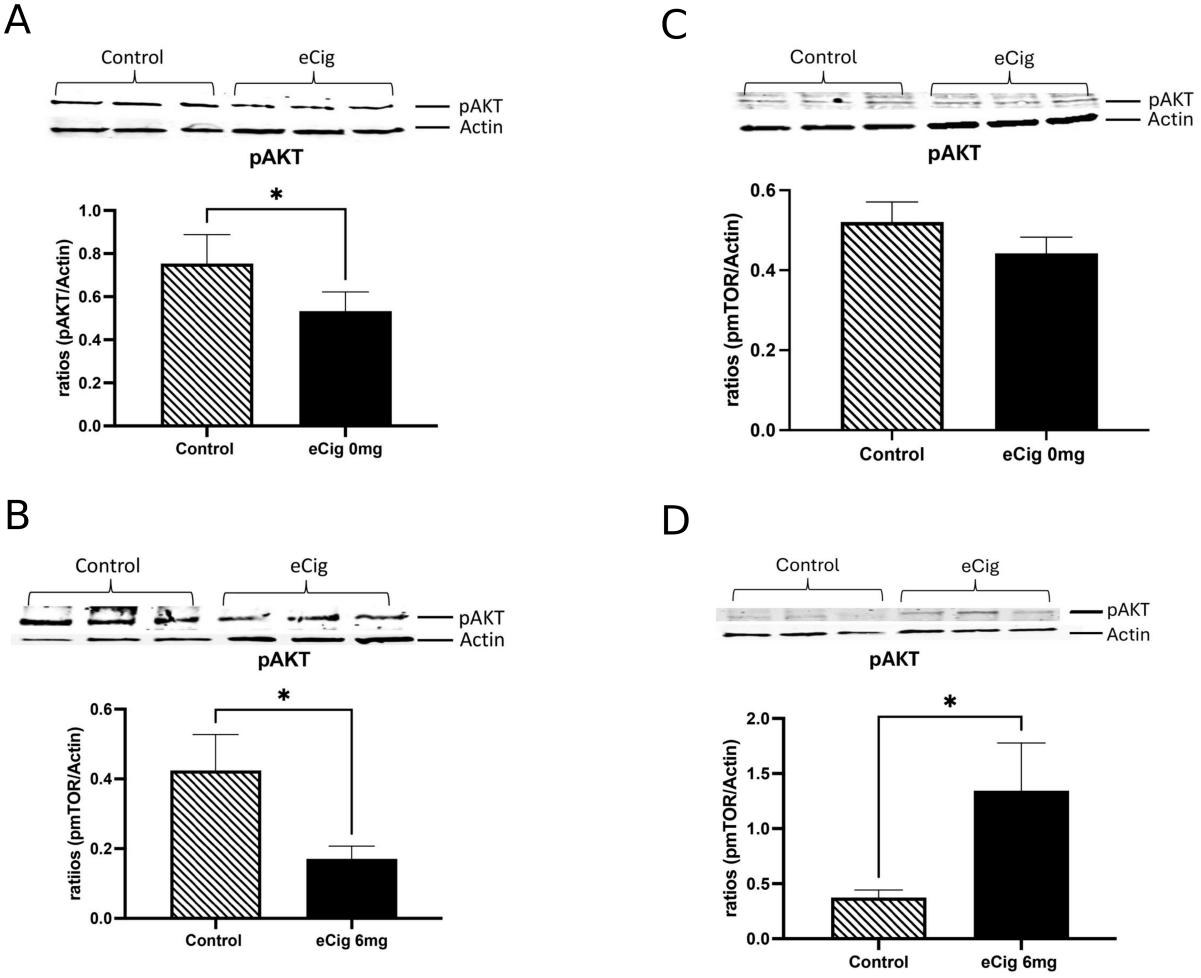
p-AKT (Ser473) in OSCC cells treated with Apple EVE. Western blot analysis and densitometric quantification of p-AKT normalized to β-actin in Ca9–22 and Cal 27 cells following 6 h exposure to 10% Apple EVE with or without nicotine (6 mg). **(A)** Ca9-22, 0 mg nicotine; **(B)** Ca9-22, 6 mg nicotine; **(C)** Cal 27, 0 mg nicotine; **(D)** Cal 27, 6 mg nicotine. Data are presented as mean ± SEM ratios of p-AKT/β-actin. *p ≤ 0.05 vs. control.

p70S6K is a kinase downstream of the mTOR signaling pathway that regulates cell growth, proliferation, and survival, and whose dysregulation is associated with cancer development and progression (12). Apple EVE without nicotine significantly increased p-p70S6K in Ca9–22 cells (1.5-fold; p<0.03) and Cal 27 cells (2.2-fold; p<0.02) compared to controls (**Figures 3A, C**). In contrast, p-p70S6K was significantly decreased in Ca9–22 cells (1.9-fold; p<0.02) treated with nicotine-containing Apple EVE, while no change was observed in Cal 27 cells (**Figures 3B, D**). The 4EBP1 protein is a key regulator of mRNA translation downstream of the mTOR pathway associated with enhanced cancer cell proliferation and tumor progression (13). There was a significant decrease in p-4EBP1 in Ca9–22 cells (3.4-fold; p<0.002), while p-4EBP1 was increased (1.9-fold; p<0.03) in Cal 27 cells following Apple EVE treatment in the absence of nicotine (**Figures 4A, B**). A significant decrease p-4EBP1 levels was detected for both Ca9-22 (3.3-fold; p<0.02) and Cal 27 (3.5-fold; p<0.03) when treated with Apple EVE and 6 mg of nicotine (**Figures 4C, D**). The AKT protein acts as a critical upstream regulator of the mTOR pathway, and its activation is frequently linked to cancer development and progression (14). p-AKT levels were decreased (1.4-fold; p<0.03) in Ca9–22 cells treated with EVE in the absence of nicotine (**Figure 5A**). In Cal 27 cells, however, p-AKT was unaffected by Apple EVE treatment compared to controls (**Figure 5B**). Treatment with Apple EVE and 6 mg nicotine decreased p-AKT in Ca9-22 (2.5-fold; p<0.02) treated with EVE and nicotine (**Figure 5C**). In contrast, Cal 27 cells showed a significant increase in p-AKT (3.6-fold; p<0.03) with this treatment (**Figure 5D**).

## 4 Discussion

Flavored electronic cigarettes (e-cigarettes), often marketed as safer alternatives to traditional tobacco, warrant closer evaluation of their biological effects on oral tissues, as suggested by our findings with Apple EVE. Emerging evidence indicates that e-cigarette aerosols, particularly those containing flavoring agents (e.g., fruit/apple flavors), can contribute to oral squamous cell carcinoma (OSCC) pathogenesis through multiple mechanisms. These include induction of oxidative stress and DNA damage (e.g., strand breaks and hypermethylation) (16), disruption of the oral microbiome leading to altered metabolic pathways associated with cancer progression, increased inflammatory cytokine release (e.g., IL-8, TNF-α), and modulation of key signaling cascades such as NF-κB, JNK, PI3K/AKT, and mTOR in oral epithelial and OSCC cells (16–20). For instance, flavored e-cigarette exposures have been linked to enhanced drug resistance via transporter alterations, pro-inflammatory and pro-senescence responses, and potential promotion of epithelial-mesenchymal transition or invasive phenotypes in head and neck cancer models. Our prior work showed that flavored e-cigarette vapor induces inflammatory and invasion-related responses in OSCC cells (1), and mTOR signaling is tightly coupled to inflammatory pathways such as NF-κB. The systemic inflammatory biomarker associations reported in head and neck disease (21, 22) are therefore consistent with, though not a direct test of, a link between e-cigarette exposure, inflammation, and the mTOR-axis changes observed here. Given the rising prevalence of e-cigarette use—especially flavored variants—among adolescents and young adults, and the perception of reduced harm compared to combustible cigarettes, elucidating their effects on cancer-related pathways in oral tissues is increasingly urgent. The mTOR pathway, a key regulator of cell growth, metabolism, and survival, is frequently dysregulated in malignancies including OSCC, positioning it as a critical target for understanding potential e-cigarette contributions to disease progression (8, 11). Nicotine has been shown to activate nicotinic acetylcholine receptors (nAChRs) expressed on epithelial and cancer cells, leading to downstream activation of PI3K/AKT signaling pathways. Consistent with this, nicotine has been reported to enhance the proliferation, migration, and invasion of head and neck squamous cell carcinoma cells, including Cal 27, through the nicotinic receptor subunit CHRNA5 and downstream MEK/ERK signaling (35). Activation of AKT can promote phosphorylation of mTOR at Ser2448 and enhance mTORC1 signaling, thereby supporting cellular survival, metabolic reprogramming, and resistance to apoptosis. In cancer cells, these pathways may be further amplified due to pre-existing oncogenic alterations, rendering malignant cells particularly responsive to nicotine-mediated signaling perturbations.

The divergent mTOR pathway responses between Ca9–22 and Cal 27 cells underscore OSCC’s intrinsic heterogeneity, stemming from differences in tissue origin, differentiation, and baseline oncogenic signaling (23). Increased p-mTOR did not uniformly activate downstream effectors (p70S6K, 4EBP1), suggesting non-canonical regulation possibly involving differential mTORC1/mTORC2 engagement or feedback inhibition (11, 24). This aligns with prior evidence of variable pathway utilization in OSCC subtypes, where dysregulated mTOR signaling—often hyperactivated in >80–90% of cases—contributes to metabolic reprogramming and sustained tumor growth (25). It is also important to recognize that both Ca9–22 and Cal 27 cells are established oral squamous cell carcinoma lines that harbor oncogenic genetic alterations, including dysregulated PI3K/AKT/mTOR signaling. Therefore, the observed changes in mTOR phosphorylation following Apple EVE exposure may reflect interactions between vapor constituents and pre-existing oncogenic signaling networks rather than *de novo* pathway induction. Malignant cells frequently exhibit constitutive growth and survival signaling, which may amplify responsiveness to nicotine-mediated stimuli. Accordingly, these findings should be interpreted within the context of cancer-specific molecular backgrounds.

While canonical mTORC1 activation promotes phosphorylation of 4EBP1 to release eIF4E and enhance cap-dependent translation and proliferation, the observed reduction in p-4EBP1 (Thr37/46) following nicotine-containing Apple EVE exposure in both Ca9–22 and CAL 27 cells may reflect context-specific regulatory mechanisms. These include negative feedback loops (e.g., S6K1-mediated inhibition of further mTORC1 activity), phosphatase activation leading to dephosphorylation, differential mTORC1/mTORC2 engagement, or direct modulation by nicotine or other vapor constituents. In certain cancer contexts, including head and neck squamous cell carcinoma, reduced 4EBP1 phosphorylation can restore its tumor-suppressive function by limiting excessive translation, potentially contributing to adaptive responses or restricted proliferation under short-term stress rather than uniform oncogenic promotion (26–29). Importantly, our study examines early signaling events (6-hour exposure) and does not assess long-term functional outcomes such as proliferation, apoptosis, or translation rates; thus, the dissociation highlights nuanced, non-canonical effects without contradicting pathway engagement by flavored e-cigarette aerosols.

Without nicotine, both cell lines showed increased p-p70S6K, whereas p-4EBP1 diverged, decreasing in Ca9–22 but increasing in Cal 27, indicating cell-type-specific downstream utilization (invasion was unchanged in both lines) (1). Interestingly, p-mTOR varied between the two OSCC cell lines: Ca9–22 cells exhibited increased p-mTOR without accompanying increases in p-p70 or p-4EBP1. In Cal 27 cells, the downstream effectors likewise did not track p-mTOR: p-4EBP1 decreased with nicotine despite elevated p-mTOR (**Figure 4D**), indicating differential, non-canonical downstream pathway utilization rather than coordinated mTORC1 activation. This suggests unique vulnerabilities to e-cigarette components across OSCC subtypes, though short-term exposure did not alter invasion in our model.

Although phosphorylation of mTOR at Ser2448 is commonly associated with mTORC1 activation, downstream substrate phosphorylation is influenced by complex regulatory mechanisms including feedback inhibition, phosphatase activity, and differential mTORC1 versus mTORC2 engagement. The dissociation observed between mTOR phosphorylation and p70S6K/4EBP1 activation may reflect cell-line–specific signaling architecture or compensatory regulatory responses to e-cigarette vapor components. These mechanisms are offered as candidate explanations consistent with the observed pattern; they were not directly tested in the present study and should be regarded as speculative rather than as established mechanistic conclusions.

Nicotine increased mTOR phosphorylation in both lines but elicited opposing effects: reduced p-AKT/p70 in Ca9–22 versus increased p-AKT/p70 in Cal 27 (15). These patterns reflect cell-specific genetic/phenotypic differences, highlighting variable responses to flavored e-cigarette aerosols.

These differential effects may be attributed to the unique genetic and phenotypic profiles of Ca9–22 and Cal 27 cells, reflecting the potential variability in OSCC’s response to e-cigarette exposure (30–32). The observed p-mTOR in response to nicotine-EVE underscores the need for further research on the longer-term biological effects of e-cigarette exposure on oral epithelial signaling. Our study demonstrates that Apple EVE, particularly in combination with nicotine, alters phosphorylation patterns of multiple components within the mTOR signaling axis in OSCC cells, with varying downstream effects on invasion and proliferation depending on the cell type.

These findings suggest that flavored e-cigarette vapor is not biologically inert (33, 34) and can perturb mTOR-associated signaling in OSCC cells (1, 20). Given the increasing prevalence of e-cigarette use, particularly among younger populations, these observations support further research into the biological effects of flavored e-cigarette aerosols on oral epithelial signaling, including the functional consequences that were not assessed in the present study.

While the present study focused on malignant OSCC cell lines, exposure of normal oral epithelial cells to e-cigarette vapor may also influence mTOR signaling and related pathways. Dysregulated activation of mTOR in non-malignant cells could theoretically contribute to altered cellular metabolism, increased proliferative signaling, or enhanced susceptibility to oncogenic transformation. However, these possibilities were not directly examined in the current study and warrant future investigation using non-transformed oral epithelial models to better define the broader carcinogenic potential of flavored e-cigarette exposure.

### 4.1 Study limitations

This study has several limitations. Only two OSCC cell lines were examined, and additional models may exhibit distinct signaling responses. Moreover, the exposure duration focused on early signaling events, which may precede later phenotypic changes. As an *in vitro* investigation, these findings may not fully reflect *in vivo* oral carcinogenesis. Nonetheless, the observed cell-line–dependent modulation of mTOR-associated signaling provides important preliminary insight into the biological effects of flavored e-cigarette vapor exposure in OSCC cells. Our findings also rest on a single technique (Western blotting); orthogonal approaches such as immunofluorescence, ELISA, kinase-activity assays, and pharmacologic pathway inhibition (e.g., rapamycin) will be needed to confirm these signaling changes. In addition, no functional endpoints were measured, and assays of proliferation, viability and cytotoxicity, apoptosis, clonogenic survival, cell-cycle distribution, and longer-duration exposures will be required to determine whether these early phosphorylation changes have downstream consequences for cell behavior. In addition, analytical quantification of nicotine concentration and chemical characterization of individual flavoring constituents were not performed and represent limitations regarding full exposure standardization. The study also did not include nicotine-only, carrier/base-liquid, or unflavored e-liquid controls, which would be needed to separate the contributions of nicotine, flavoring agents, and carrier components. The present design instead isolates the effect of nicotine within a marketed flavored product by comparing Apple EVE with and without nicotine against untreated cells, and constituent-level deconvolution together with chemical characterization of the aerosol are planned as next steps.

In addition, the study focused on phosphorylation status of key signaling proteins without parallel assessment of total protein expression levels. Although normalization to β-actin was performed to control for loading variability, the absence of total protein measurements limits the ability to determine whether observed changes reflect altered phosphorylation dynamics alone or potential variations in total protein abundance. Accordingly, we do not interpret these results as definitive evidence of altered pathway activity, and normalization of phosphorylated proteins to their total (non-phosphorylated) forms is a priority for future work. In particular, we cannot exclude that some of the observed differences reflect changes in total protein expression rather than site-specific phosphorylation.

Although minor variability in β-actin signal intensity was observed, normalization was applied consistently across independent experiments, minimizing the likelihood of systematic bias.

## 5 Conclusions

This study demonstrates that Apple EVE, particularly in the presence of nicotine, induces cell-line-specific alterations in the phosphorylation of key mTOR pathway proteins (mTOR, p70S6K, 4EBP1, and AKT) in OSCC cell lines Ca9–22 and Cal 27. While no changes in cell invasion were observed, the divergent signaling responses—such as increased p-mTOR in both lines with nicotine, but opposing effects on downstream effectors—highlight the potential for flavored e-cigarette aerosols to modulate cancer-associated pathways in a heterogeneous manner reflective of OSCC variability. Because invasion was unchanged and no proliferative, apoptotic, or other functional endpoint was assessed, the biological significance of these phosphorylation changes for cell behavior remains to be determined.

These findings suggest that flavored e-cigarettes are not biologically inert and can perturb mTOR-related signaling in OSCC cells, which warrants continued attention given their growing use, especially among younger populations. However, as an *in vitro* investigation focused on early events, results should be interpreted cautiously. As this study examined short-term signaling responses, functional consequences such as proliferation, apoptosis, or migration remain to be determined in future studies. Future research should incorporate additional cell lines, longer exposures, *in vivo* models, and multi-omics approaches to clarify the longer-term biological implications of flavored e-cigarette exposure for oral epithelial cells.

## Data Availability

The original contributions presented in the study are included in the article. Further inquiries can be directed to the corresponding author.
